# Identification of Chinese Herbal Medicines from *Zingiberaceae* Family Using Feature Extraction and Cascade Classifier Based on Response Signals from E-Nose

**DOI:** 10.1155/2014/963035

**Published:** 2014-06-19

**Authors:** Lian Peng, Hui-Qin Zou, Rudolf Bauer, Yong Liu, Ou Tao, Su-Rong Yan, Yu Han, Jia-Hui Li, Zhi-Yu Ren, Yong-Hong Yan

**Affiliations:** ^1^School of Chinese Materia Medica, Beijing University of Chinese Medicine, No. 6 Wang Jing Zhong Huan Nan Lu, Chao Yang District, Beijing 100102, China; ^2^Library, Beijing University of Chinese Medicine, No. 11 Bei San Huan Dong Lu, Chao Yang District, Beijing 100029, China; ^3^Institute of Pharmaceutical Science, University of Graz, Graz 8010, Austria

## Abstract

Identification of Chinese herbal medicines (CHMs) by human experience is often inaccurate because individual ability and external factors may influence the outcome. However, it might be promising to employ an electronic nose (E-nose) to identify them. This paper presents a rapid and reliable method for identification of ten different species of CHMs from *Zingiberaceae* family based on their response signals from E-nose. Ten* Zingiberaceae *CHMs were measured and their maximum response values were analyzed by principal component analysis (PCA). Result shows that *E Zhu* (*Curcuma phaeocaulis* Val.) and * Yi Zhi* (*Alpinia oxyphylla* Miq.) could not be distinguished completely by PCA. Two solutions were proposed: (i) using BestFirst+CfsSubsetEval (BC) method to extract more discriminative features to select sensors with higher contribution rate and remove the redundant signals; (ii) employing a novel cascade classifier with two stages to enhance the distinguishing-positive rate (DPR). Based on these strategies, six features were extracted and used in different stages of the cascade classifier with higher DPRs.

## 1. Introduction

Chinese herbal medicines (CHMs) are getting more and more international attention owing to their in vivo antitumor activities [1], alternative treatment for menopausal hot flushes [2], inhibition of cancer-related inflammation [3], and so on. Accurate medication of CHMs is an important issue as their global use is increasing rapidly. Besides, incorrect, inferior, or fake CHMs may result in poor clinical effects or even poisoning [4]. Therefore, it is urgent and necessary to establish rapid, accurate, and convenient methods for CHMs identification.

Classically, CHMs are authenticated by a human experience panel called macroscopic identification, which considers the morphologic characteristics (shape, color, etc.) and the organoleptic properties (odor, taste, etc.) through observing, touching, smelling, and tasting. In* Pharmacopoeia of the People's Republic of China*, this macroscopic identification is still an important aspect of CHMs quality evaluation. For instance,* Sha Ren* (dried fructus of* Amomum villosum* Lour.) with large size and strong odor should be superior in quality. However, this method is expensive and time-consuming and also inaccurate because of its low sensitivity and lack of quantitative information. Electronic nose (E-nose) is a rapid, reliable, and robust technology and could be employed as easy-to-use and cost-efficient technology.

In the past decades, E-nose has been applied in many fields [5]. Also, many studies on application of E-nose to quality control of CHMs and food are available in the literatures [6, 7].

Yu and Wang [8] considered the volume of vial and the headspace generated time relating to the identification results. However, almost no studies have been reported on the orthogonal analysis of experimental factors. In a recent study, Ye et al. [9] employed principal component analysis (PCA) to construct a discriminative model based on E-nose to successfully distinguish odors of authentic, fake, and adulterate* musk* samples. However, most of them construct the discriminative model with only one single kind of classifier such as ANN, RBF, and so on. Only few studies on application of E-nose with cascade classifier for image recognition [10] are reported. In this paper, orthogonal test is designed to determine the optimum experimental procedure and two new solutions are proposed to improve the distinguishing-positive rate (DPR) for classifying different CHMs from* Zingiberaceae* family.

As is well-known, CHMs from* Zingiberaceae* family share some chemical components together such as eucalyptol, zingiberene, and camphor. Hence some of them have quite similar odor. Nevertheless, some subtle differences among the odors of different CHMs exist according to the unique chemical component and the different proportions of chemical composition. But these differences are very small and their effects on the odors could be distinguished only by well-trained people. The employment of E-nose with optimum experimental procedure and classifiers could be useful for identifying different* Zingiberaceae* CHMs.

## 2. Subjects and Methods

### 2.1. Subjects

The research was carried out by using ten different kinds of* Zingiberaceae* CHMs, which were obtained from Beijing Tongrentang Co., Ltd. (Beijing, China) and authenticated by Professor Yonghong Yan in Beijing University of Chinese Medicine (Beijing, China). As illustrated in Table 1, samples were labeled as* Jiang Huang, Gan Jiang, Gao Liang Jiang, E Zhu, Yu Jin, Bai Dou Kou, Cao Dou Kou, Cao Guo, Yi Zhi, *and* Sha Ren*.

### 2.2. E-Nose

Experiments were performed by *α*-FOX3000 E-nose operating with the enrichment and desorption detector unit. The system was from Alpha M.O.S. (France). Also *α*-FOX3000 E-nose consists of air generator equipment, a sampling apparatus, HS-100 autosampler, a detector unit, and a computer for data recording. The detector unit is composed of 12 metal oxide sensors (MOSs), shown in Table 2. The sensor response represents the conductance ratio (*G*_0_ */G*).

Grinded into small particles, a certain amount of each sample was accurately weighed into a 10 mL septa-sealed bottle and loaded into the autosampler tray with a rotation rate of 250 rpm. After incubation, 500 *μ*L of headspace air was automatically injected into the detector unit with a constant rate of 500 *μ*L/s through a syringe. The conductance ratio of each sensor changed during the measurement process, which was recorded by a computer program. The measurement phase went on for 200 s, which was enough for all the sensors to reach the stable values and return to the baseline. The data acquisition cycle was 1 s and the responses were collected in the computer for later use.

### 2.3. Optimization of Experimental Conditions by Orthogonal Test Design

The quantity of each sample was selected from 0.1 g, 0.2 g, 0.3 g, 0.4 g, 0.5 g, and 1 g. Based on the selected quantity, an orthogonal array (L_9_(3^3^)) was constructed to evaluate the effects of the following factors: particle size (A), incubation temperature (B), and incubation time (C). Factors are displayed in Table 3. Each experiment was performed in triplicate and the data were analyzed using variance analysis (ANOVA). Based on the results, the optimum experiment procedure was determined.

Twelve repeated samples were prepared for each kind of CHMs and totally 120 measurements were performed by the optimum experiment procedure. The E-nose response values of CHMs' samples were recorded and classifiers were established to identify them. All the samples were measured by dynamic headspace sampling.

### 2.4. Principal Component Analysis, Radial Basis Function, and Random Forests

The data attained by the process mentioned above was analyzed by different pattern recognition techniques including principal component analysis (PCA), radial basis function (RBF), and random forests (RF) to construct different classifiers.

Tenfold cross validation and external test set validation were used to evaluate the performance of different classifiers with DPR. The classification results should not be accepted if the DPR was lower than 80%.

PCA is a projection method that provides easy visualization of all the information contained in a dataset [11]. Besides, PCA helps us figure out which samples are different from others and which principal component extracted from the original variances contributes more to this difference.

RBF is a kind of artificial neural networks which have simple topological structure and clear learn procedure. RBF has its unique advantages in pattern recognition application due to its quick convergence speed and having no local minima [12]. After training, RBF can provide us with a discriminative model for blind samples analyses.

RF technique is a novel classifier containing multiple decision trees for analysis of high dimension data which is able to exploit dependencies and structures contained within spatially varying input data [13]. Self-learning ability of RF helps us avoid misjudgment and improve accuracy.

While screening the suitable neural networks or decision trees in the classifiers system, the system processes the inputs and compares its resulting outputs to the desired outputs. Errors are propagated back to the system and tell the system to adjust its inner structures so as to improve the distinguishing-positive rate. During the training, the same dataset will be processed many times as the connection weights are refined.

### 2.5. Feature Extraction by BestFirst+CfsSubsetEval and Cascade Classifier Analysis

Feature extraction analysis aims to screen all the variances, selecting the factors with more valuable information to the final target and eliminating others with redundant information, which can decrease high dimensions into low dimensions and help the classifier get more accurate results. BestFirst+CfsSubsetEval (BC) is one kind of feature extraction technologies. It can screen out the characteristic parameter vectors with high relevance to the classification. We can get an optimum set of sensors for final identification based on BC.

Cascade classifier contains more than one kind of pattern recognition algorithms, which can improve the identification ability of the system and increase the DPR [14]. This paper presents a two-stage cascade classifier with RBF and RF for distinguishing those different CHMs from* Zingiberaceae* family.

**Figure 1:**
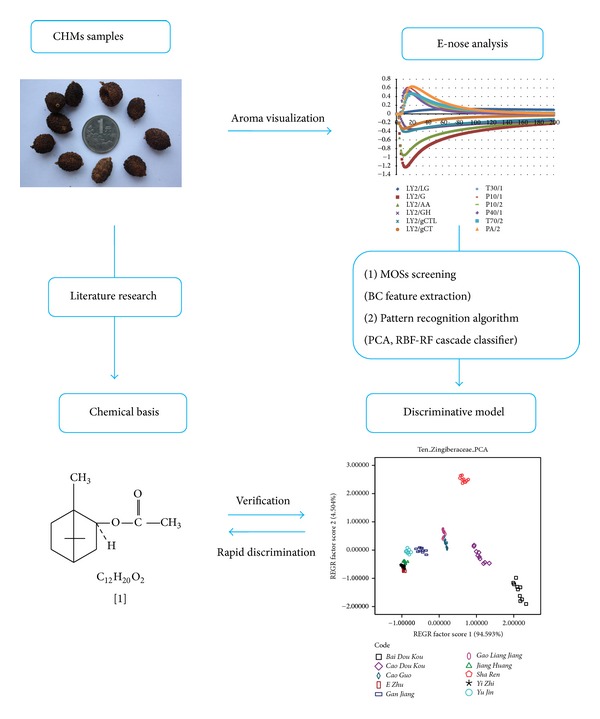
Scheme of the work flow.

Figure 1 shows the scheme of the work flow.

## 3. Results and Discussion

### 3.1. E-Nose Responses to CHMs Aroma

When estimating the sensor response to a given sample, the response values were used as follows: *R* = *G*_0_/*G*, where *R* was the response, *G*_0_ was the conductance of a sensor in the reference air, and *G* was the conductance of the sensor in the sample gas.

**Figure 2:**
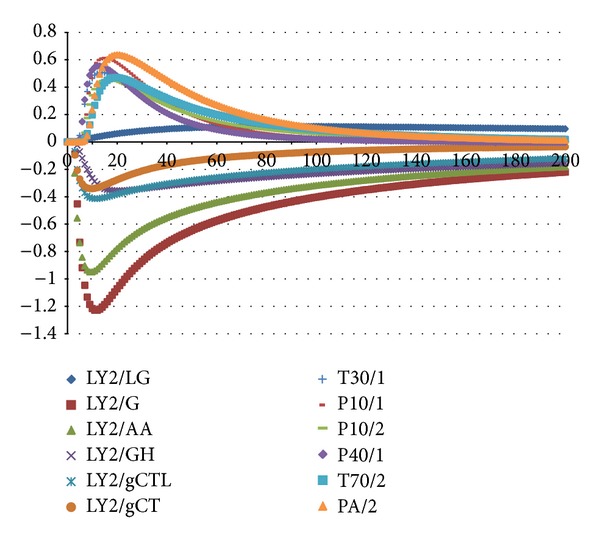
Typical responses of 12 MOSs measuring a* Sha Ren* sample.

Figure 2 shows typical responses of twelve MOSs measuring a* Sha Ren* sample. One line represents one MOS's signals measuring the* Sha Ren* sample. The horizontal axis is the timeline, a total of 200 seconds; the vertical axis is the response values of the MOS. The curves represent the resistance value of each sensor against time due to the electrovalve action when the volatile compounds reached the detection chamber. In the initial period, the response value of each MOS is low and then increases continuously and finally stabilizes after a few seconds. In this study, 12 maximum response values of each CHM sample from 12 MOSs were extracted and analyzed individually.

The repeatability of the established method was evaluated with nine parallel tests of CHMs' samples. The relative standard deviation (RSD, *n* = 6) values of 12 MOSs were calculated. The results are all less than 3%, proving a high repeatability of MOSs responses.

### 3.2. Variance Analysis (ANOVA)

**Figure 3:**
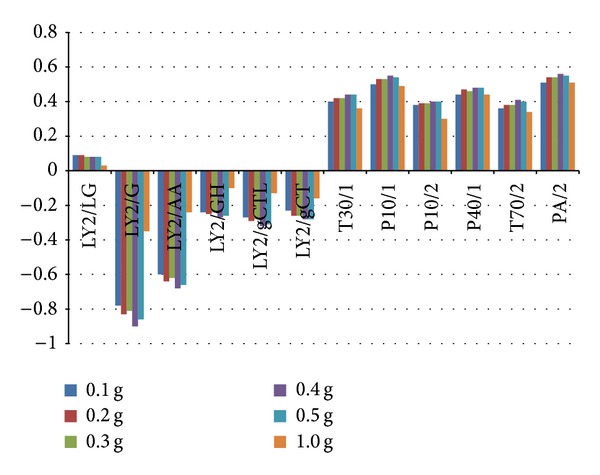
Responses of 12 MOSs measuring* Sha Ren* samples of 0.1 g, 0.2 g, 0.3 g, 0.4 g, 0.5 g, and 1 g.

The amount of a sample is important for the detection of CHMs aroma and affects the volume and the concentration of the headspace gas because the sample is loaded into a 10 mL septa-sealed bottle. To select a suitable sample quantity, experiments with 0.1 g, 0.2 g, 0.3 g, 0.4 g, 0.5 g, and 1 g samples were performed while the other experiment conditions were the same.  Figure 3 shows that in the beginning the responses of 12 MOSs increase with more amounts of samples from 0.1 g to 0.4 g. Then the responses decrease a little bit with 0.5 g and 1 g compared to 0.4 g. Besides, overload occurs in the system while measuring 1 g samples because the loading capacity and the absorption rate of the MOSs are limited. Overload could result in the problem of poisoning sensor. So 0.4 g samples were determined to obtain higher responses of the MOSs and protect them from being poisoned.

**Figure 4:**
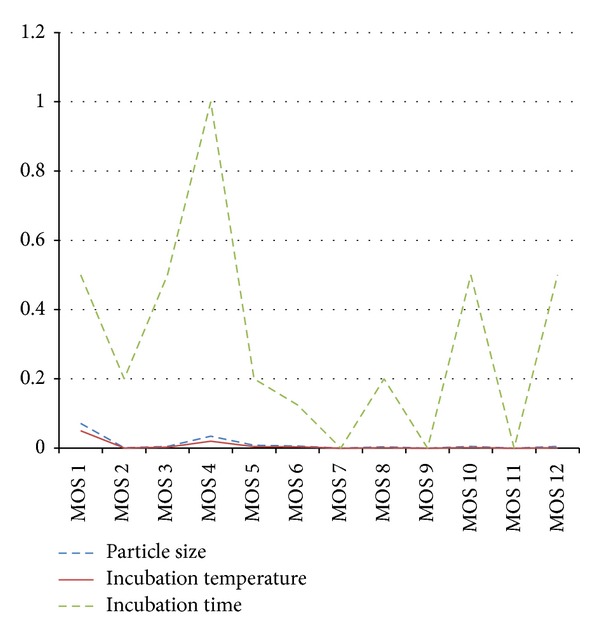
Results of variance analysis for the orthogonal test (*Sha Ren*).

In order to evaluate the effects of the particle size of the samples (A: 850 ± 29 *μ*m, 355 ± 13 *μ*m, and 250 ± 9.9 *μ*m), the incubation temperature (B: 35°C, 40°C, and 45°C), and the incubation time (C: 360 s, 480 s, and 600 s) on the responses of 12 MOSs, variance analysis (ANOVA) was performed based on the maximum values of 12 MOSs responses. The results are illustrated in Table 4 and Figure 4 (samples of* Sha Ren*). The magnitudes of the* Pr*-values in Table 4 and Figure 4 indicate the relative importance of the three factors. The particle size and incubation temperature have significant effects on the responses of 11 MOSs (MOS 2–MOS 12); however, the incubation time has significant effect on the responses of 3 MOSs only. Besides, relative standard deviation (RSD) values of different incubation temperatures with six repeated samples for each experiment show that incubation temperature of 45°C has the lowest and most stable RSD value (all of them less than 2%). In order to obtain more stable responses, the CHMs' samples were measured after incubating at 45°C. The RSD analyses of the other two factors show that particle size of 850 ± 29 *μ*m and incubation time of 600 seconds are the optimum experiment conditions.

### 3.3. Pattern Recognition (PCA, RBF, and RF)

**Figure 5:**
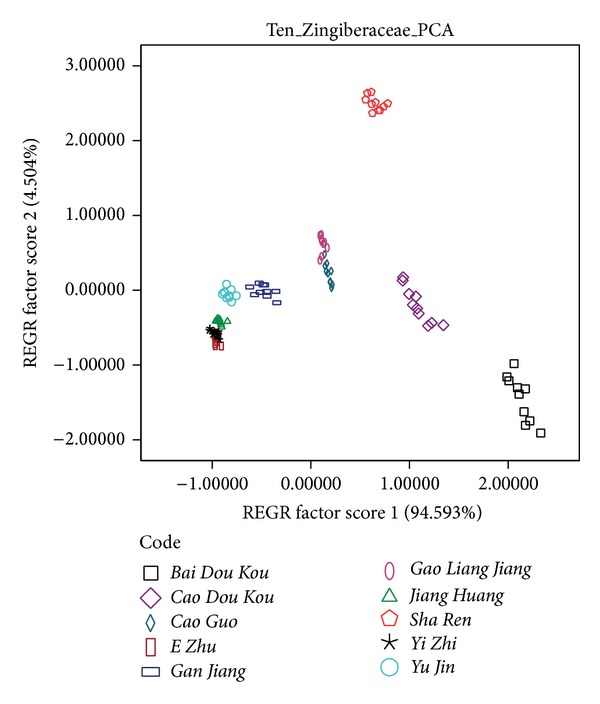
Result of ten* Zingiberaceae* CHMs by PCA.

The dataset obtained from ten kinds of CHMs' samples was analyzed by PCA. The PCA plot is shown in Figure 5. Most of the samples could be classified by PCA. These CHMs share the same characteristic that they all have one or more diagnostic chemical compounds such as* Sha Ren which* contains bornyl acetate,* Bai Dou Kou* which contains clove dilute, and* Cao Dou Kou* which contains humulene. These unique chemical compounds affect their aroma and make them easier to be identified. However, some samples of* E Zhu* and* Yi Zhi *are overlapped owing to their similar chemical composition, for instance, eucalyptol. It is more difficult to identify them than others. Although these ten species of* Zingiberaceae *CHMs could not be completely identified by PCA, the results are in accordance with the identification result by human experience. As shown in Figure 5, aroma differences among* Sha Ren, Cao Dou Kou, Bai Dou Kou, and Gan Jiang* are obvious and the aroma of* Yi Zhi* is close to that of* E Zhu*.

In further study, RBF and RF were applied to establish classifiers. The 120 samples (12 duplicates for each CHM) were divided into two groups: 100 samples (10 of each CHM) for the training set and the remaining 20 samples (2 of each CHM) for the test set. Most of the datasets were taken as training set in order to get better classifier after training. As for the training set, the input vectors were 12 dimensions from the maximum values of the 12 MOSs. The output vectors were determined to be ten dimensions corresponding to ten species of CHMs. The identification result was evaluated by two validation methods including tenfold cross validation in the training set and external test set validation in the test set and DPRs were calculated. The results are shown in Table 5. The DPRs are all above 80%, so the identification result can be accepted. But the DPRs in the test set are just 80%. More detailed identification results by RBF are shown in Table 6. It tells us that the identification errors mostly exist between* E Zhu* and* Yi Zhi*. Since they share more chemical characteristics, the difference of them is subtle. It is necessary to apply other data mining technologies to improve the classifier.

### 3.4. Optimization of Discriminative Model by Feature Extraction and Cascade Classifier

Six sensors (MOS 2, MOS 3, MOS 6, MOS 8, MOS 10, and MOS 12) with more valuable information for distinguishing the samples were selected by BA feature extraction analysis. The result shows that DPR in the test set by RBF increases from 80% to 85%.

In this study, a two-stage cascade was constructed focusing on the identification of* E Zhu* and* Yi Zhi*. Dataset-*φ*1 was established by each kind of CHMs as one category used in stage I classifier of RBF. And dataset-*φ*2 was established by* E Zhu*,* Yi Zhi,* and the remaining eight kinds of CHMs as three different categories, respectively, used in stage II classifier of RF. Detailed results are as follows: a higher DPR of 90% instead of 70% for* E Zhu *while being 80% instead of 70% for* Yi Zhi* by tenfold cross validation in stage II. All the CHMs' samples could be distinguished with high DPRs by this two-stage cascade classifier.

## 4. Conclusions

The results of ANOVA and multivariance analysis showed that the suitable quantity of sample for measurement is 0.4 g and the particle size and incubation temperature mainly influence the responses of most MOSs (MOS 2–MOS 12). Based on orthogonal test design, the optimum experimental conditions were determined: particle size of 850 ± 29 *μ*m, incubation temperature of 45°C, and incubation time of 600 seconds.

The responses of* E Zhu* and* Yi Zhi* are partly overlapped and thus these two CHMs' samples could not be identified completely by PCA. In accordance with the results by human experience, the aroma detected by human nose and E-nose is more different among* Sha Ren*,* Bai Dou Kou,* and* Cao Dou Kou*.

The classification results of RBF and RF analysis were superior to that of PCA. All the DPRs were above 80% and the CHMs' samples from* Zingiberaceae* family could be classified. However, some samples of* E Zhu* were misjudged as* Yi Zhi* owing to their similar chemical compositions and sensual scent.

Two solutions, BC feature extraction and two-stage cascade classifier, were proposed to improve the identification ability of the discriminative model, via removing redundant information to reduce the data dimensions and to separate similar and difficult-to-distinguish samples from others. Results showed that both solutions were feasible and the DPRs were increased: a higher DPR of 85% instead of 80% by external test set validation in stage I; a higher DPR of 90% instead of 70% for* E Zhu *while being 80% instead of 70% for* Yi Zhi* by tenfold cross validation in stage II.

## Figures and Tables

**Table 1:** *Zingiberaceae* plant as a herbal medicine.

Number	Label	Plant as a herbal medicine
1	*Jiang Huang *	Rhizoma of* Curcuma longa* L.
2	*Gan Jiang *	Rhizoma of *Zingiber officinale* Rosc.
3	*Gao Liang Jiang *	Rhizoma of *Alpinia officinarum* Hance
4	*E Zhu *	Rhizoma of *Curcuma phaeocaulis* Val.
5	*Yu Jin *	Radix of *Curcuma kwangsiensis* S. G. Lee et C. F. Liang
6	*Bai Dou Kou *	Fructus of *Amomum kravanh* Pierre ex Gagnep.
7	*Cao Dou Kou *	Semen of *Alpinia katsumadai* Hayata
8	*Cao Guo *	Fructus of *Amomum tsaoko* Crevost et Lemaire
9	*Yi Zhi *	Fructus of *Alpinia oxyphylla* Miq.
10	*Sha Ren *	Fructus of *Amomum villosum* Lour.

**Table 2:** Response feature of the sensor array.

Number in array	Sensor name	Object substance for sensing
MOS 1	LY2/LG	Oxidizing gas
MOS 2	LY2/G	Ammonia, carbon monoxide
MOS 3	LY2/AA	Ethanol
MOS 4	LY2/GH	Ammonia/organic amine
MOS 5	LY2/gCTL	Hydrogen sulfide
MOS 6	LY2/gCT	Propane/butane
MOS 7	T30/1	Organic solvents
MOS 8	P10/1	Hydrocarbons
MOS 9	P10/2	Methane
MOS 10	P40/1	Fluorine
MOS 11	T70/2	Aromatic compounds
MOS 12	PA/2	Ethanol, ammonia/organic amine

**Table 3:** Orthogonal design factors and levels.

Factors	Level		
	1	2	3
A: particle size	850 ± 29 *μ*m	355 ± 13 *μ*m	250 ± 9.9 *μ*m
B: incubation temperature	35°C	40°C	45°C
C: incubation time	360 s	480 s	600 s

**Table 4:** Results of variance analysis for the orthogonal test (*Sha Ren*).

Pr	A: particle size	B: incubation temperature	C: incubation time
MOS 1	0.0714	0.0500	0.5000
MOS 2	0.0009∗	0.0005∗	0.2000
MOS 3	0.0050∗	0.0029∗	0.5000
MOS 4	0.0345∗	0.0200∗	1.0000
MOS 5	0.0080∗	0.0041∗	0.2000
MOS 6	0.0063∗	0.0037∗	0.1250
MOS 7	<0.0001∗	<0.0001∗	<0.0001∗
MOS 8	0.0037∗	0.0012∗	0.2000
MOS 9	<0.0001∗	<0.0001∗	<0.0001∗
MOS 10	0.0050∗	0.0014∗	0.5000
MOS 11	<0.0001∗	<0.0001∗	<0.0001∗
MOS 12	0.0048∗	0.0010∗	0.5000

*Significant at the probability *P* ≤ 0.05 level.

**Table 5:** Results of RBF and RF analyses.

Classifier	Tenfold cross validation	External test set validation
RBF	96%	80%
RF	94%	80%

**Table 6:** Detailed results of RBF analysis.

Classification as→	a	b	c	d	e	f	g	h	i	j
a [1 0 0 0 0 0 0 0 0 0]	10	0	0	0	0	0	0	0	0	0
b [0 1 0 0 0 0 0 0 0 0]	0	10	0	0	0	0	0	0	0	0
c [0 0 1 0 0 0 0 0 0 0]	0	0	10	0	0	0	0	0	0	0
d [0 0 0 1 0 0 0 0 0 0]	0	0	0	9	0	0	0	0	1	0
e [0 0 0 0 1 0 0 0 0 0]	0	0	0	0	10	0	0	0	0	0
f [0 0 0 0 0 1 0 0 0 0]	0	0	0	0	0	10	0	0	0	0
g [0 0 0 0 0 0 1 0 0 0]	0	0	0	0	0	0	9	0	1	0
h [0 0 0 0 0 0 0 1 0 0]	0	0	0	0	0	0	0	10	0	0
i [0 0 0 0 0 0 0 0 1 0]	0	0	0	2∗	0	0	0	0	8	0
j [0 0 0 0 0 0 0 0 0 1]	0	0	0	0	0	0	0	0	0	10

Note: a: *Jiang Huang*; b: *Gan Jiang*; c: *Gao Liang Jiang*; d: *E Zhu*; e: *Yu Jin*; f: *Bai Dou Kou*; g: *Cao Dou Kou*; h: *Cao Guo*; i: *Yi Zhi*; j: *Sha Ren*.

*Two samples of *Yi Zhi* were misjudged as *E Zhu*.

## Abbreviations

CHM: Chinese herbal medicine
E-nose: Electronic nose
MOS: Metal oxide semiconductor
PCA: Principal component analysis
BC: BestFirst+CfsSubsetEval
RBF: Radial basis function
RF: Random forests
DPR: Distinguishing-positive rate.
